# Mapping the Evidence on Food Security Outcomes and Initiatives Among Climate Refugees: A Scoping Review

**DOI:** 10.3390/foods15040777

**Published:** 2026-02-21

**Authors:** Odette Wills, MacKenzie Kerr, Mohammad Reza Pakravan-Charvadeh, Zoe Longworth, Mojtaba Shafiee, Hassan Vatanparast

**Affiliations:** 1 School of Public Health, University of Saskatchewan, Saskatoon, SK S7N 5E5, Canada; oaw820@mail.usask.ca; 2 College of Pharmacy and Nutrition, University of Saskatchewan, Saskatoon, SK S7N 5E5, Canada; mlk649@mail.usask.ca (M.K.); zoe.longworth@usask.ca (Z.L.); 3 Department of Agricultural Economics and Rural Development, Faculty of Agriculture, Lorestan University, Khorramabad 68137-17133, Iran; pakravan.m@lu.ac.ir; 4 Margaret Ritchie School of Family and Consumer Sciences, College of Agricultural and Life Sciences, University of Idaho, Moscow, ID 83844, USA

**Keywords:** climate change, food security, migration, refugees, food security initiatives

## Abstract

The increasing severity of climate change poses profound challenges to global food security, particularly affecting vulnerable populations such as migrants and refugees. This scoping review examines the nexus between climate change, food security, and migration, focusing on the impacts and responses within affected communities. Guided by the Preferred Reporting Items for Systematic Reviews and Meta-Analyses Extension for Scoping Reviews (PRISMA-ScR), this review synthesized literature across multiple databases, including Ovid MEDLINE, Embase, Global Health, Public Health, Web of Science, and PsycINFO. The search yielded 908 records, with nine articles meeting the inclusion criteria. Across studies, climate-related stressors such as rainfall variability, flooding, and drought were consistently linked to livelihood disruption and food insecurity, often shaping migration and displacement decisions. However, food security outcomes were defined and measured inconsistently, ranging from crop yields and food availability to coping strategies and self-reported hunger, limiting comparability across studies. Evidence on food security initiatives was largely descriptive, with few studies assessing intervention effectiveness or post-displacement food security outcomes. Overall, the mapped literature emphasizes food insecurity as a key mediating pathway between climate change and mobility, but reveals important gaps related to standardized outcome measures, evaluation of food security initiatives, and the food security experiences of displaced populations at destination.

## 1. Introduction

Climate change historically includes variations in the global climate due to natural factors such as slight changes in Earth’s orbit and variations in solar energy [1,2]. However, the current accelerated rate of climate change is predominantly attributed to human activities, particularly the emissions of greenhouse gases [1,3,4]. Climate change has contributed to more frequent and severe weather events, including excessive rainfall and prolonged periods of intense dryness. These climatic shifts have had profound effects on human lifestyles, notably forcing a substantial number of individuals to become ‘climate migrants’ or ‘climate refugees.’ These individuals are forced to leave their homes due to severe climate events, facing insecurity and seeking asylum in other regions or countries [5,6]. Once relocated, they must rebuild their lives under challenging conditions [5,6]. In 2022 alone, disasters triggered a record 32.6 million internal displacements, with 98% of these movements predominantly caused by weather-related events such as floods, storms, wildfires, and droughts [7]. This figure represents a 41% increase in weather-related displacements compared to 2008, underscoring the escalating impact of climate change on human mobility [7].

The relationship between climate change and human displacement has a historical dimension that is interconnected with agricultural practices and food cycles [8]. For instance, extreme droughts or floods can devastate crop yields, threatening the food security of rural populations dependent on agriculture [9]. Moreover, as global temperatures continue to rise, sustainable food production faces significant challenges, including decreased soil fertility and the destruction of crops due to floods or storms [10]. While this climate–food–migration linkage is widely recognized, much of the existing literature emphasizes broad causal narratives [8,11] rather than systematically examining how food security is addressed before, during, and after displacement. As a result, the mechanisms through which food insecurity shapes displacement outcomes, and the effectiveness of food security initiatives in these contexts, remain insufficiently synthesized.

Food security is achieved when individuals consistently have access to safe and nutritious food that is culturally appropriate and supports their lifestyle [12,13]. This concept is founded on four pillars: availability, accessibility, utilization, and stability [14], each critical to ensuring that displaced populations can maintain a healthy diet despite the challenges they face. Predominantly, the adverse effects of climate change will most heavily affect impoverished regions, predominantly found in the Global South [15]. This region includes Africa, Southeast Asia, the Middle East, Mexico, and Central and South America, aligning mostly with the world’s less developed and economically disadvantaged areas. The World Bank highlights that in developing countries, disruptions in food intake and eating patterns constitute major health challenges [16,17]. Approximately 690 million people, or 8.9% of the population in these regions, are food insecure [16,17]. Some host countries and aid agencies provide food as initiatives for refugees, but resources are often stretched thin, which jeopardizes sustainable and consistent access to food for these vulnerable populations [13]. Despite growing attention to climate-induced migration, there remains a fragmented understanding of how food security initiatives are incorporated into climate adaptation and displacement responses [18,19].

This scoping review responds to these gaps by mapping the available literature on climate change, food security initiatives, and food security outcomes among displaced populations, with the aim of identifying where evidence converges, where it diverges, and where future research is most urgently needed. More specifically, this study also identifies the changes in the food security status of resultant migrants. It aims to capture food security initiatives such as humanitarian aid agencies, community gardens and kitchens, and cooking programs. Given the expected increase in climate-induced migration, this review underscores the urgent need to explore sustainable initiatives that support not only human populations but all living systems on the planet.

## 2. Materials and Methods

For this scoping review, we adhered to the Preferred Reporting Items for Systematic Reviews and Meta-Analyses Extension for Scoping Reviews (PRISMA-ScR) to ensure a rigorous and transparent documentation. The PRISMA-ScR guidelines, which are specifically designed to guide the reporting of scoping reviews, are publicly accessible online. Additional details on the methodology and scoping review protocol were derived from a foundational article by Tricco et al. [20], which provides further guidance on implementing these guidelines effectively. A review protocol was developed a priori but was not registered or published.

### 2.1. Eligibility Criteria

Articles were eligible for inclusion if they examined links between “climate change”, “food security”, and “human mobility”, with particular attention to displaced populations, including refugees, internally displaced persons, and climate-related migrants. Food security was defined broadly to capture multiple dimensions, including food availability, access, utilization, stability, coping strategies, and food-related livelihoods. This inclusive definition was intentionally adopted to reflect the heterogeneity of how food security is conceptualized and measured in the existing literature. Climate change was operationalized to include both slow-onset processes such as rainfall variability and drought, and rapid-onset events such as floods and extreme weather. Displacement was defined to include internal migration and displacement driven wholly or partly by climate-related stressors, recognizing that legal and conceptual distinctions between migrants and refugees are not consistently applied across studies.

Exclusion criteria included articles not available in English, review-style articles, and/or those failing to address any of the key descriptors. Throughout the systematic process, the authors also encountered news articles and conference publications: these were systematically excluded from the review due to their typically brief content and the potential for bias, as well as the lack of comprehensive research backing. While the use of broad categories limited direct comparability across studies, this approach was considered appropriate for a scoping review, where the objective is to map how key concepts are defined, applied, and operationalized across the evidence base rather than to assess intervention effectiveness. This decision informed the interpretive stance of the review, with findings synthesized descriptively and analytically rather than through direct comparison of outcomes.

### 2.2. Information Sources and Article Selection

The search for relevant literature was conducted on 2 August 2023, across multiple databases: Ovid MEDLINE, Embase, Global Health, Public Health, Web of Science, and PsycINFO. The search strategy was developed with the guidance of a Health Sciences librarian from the University of Saskatchewan and refined through discussions with all authors. An illustrative example of a search in Embase is provided in Table A1. The literature searches were executed similarly across all databases. Results from these searches were consolidated using Rayyan software^©^ (Rayyan Systems Inc., Cambridge, MA, USA), where duplicates were removed. To ensure thorough coverage, backward citation chaining was employed, and bibliographies of included papers were examined to identify additional relevant articles. The initial searches and systematic screening were primarily conducted by the first author (O.W.), with the second author (M.K.) assisting in the screening process. Discrepancies regarding article inclusion were resolved through consultation with additional authors (Z.L.L. and H.V.).

**Figure 1:**
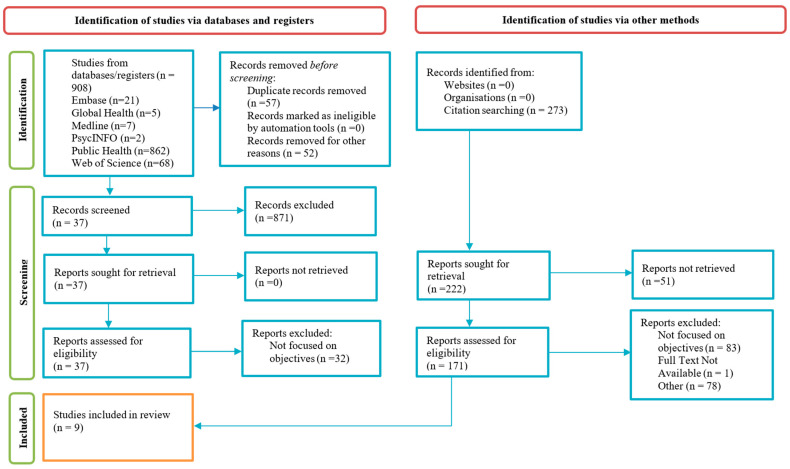
Preferred Reporting Items for Systematic Reviews and Meta-Analyses (PRISMA) reporting flow diagram.

The search from all the databases yielded 908 records—Medline (*n* = 7), Embase (*n* = 21), Global Health (*n* = 5), Public Health (*n* = 862), Web of Science (*n* = 68), and PsychInfo (*n* = 2). A total of 57 duplicates were discovered and removed, resulting in 851 sources available for screening. Initial screening of titles and abstracts led to the exclusion of 814 records due to ineligibility, leaving 37 articles for full-text review. Of these, only five articles met the study objectives upon detailed examination. To further supplement the included sources, the authors completed backward citation chaining, where 273 additional sources underwent screening, and four sources met the inclusion criteria. Common reasons for exclusion included the lack of discussion on all relevant keywords and/or concepts. For instance, some studies focused on migration due to socio-economic grounds; others focused on mental health instead of food security. Ultimately, through database searches and backward citation chaining, nine articles were selected for inclusion. This selection process, following the PRISMA guidelines, is detailed in Figure 1. All authors concurred on the final list of included articles.

### 2.3. Data Charting Procedure

To systematically extract and organize the relevant data from the included articles, Table 1 was developed. This table includes several key data points: author(s) and year of publication, study aims, study design, along with population and location details, specific climate change events studied (e.g., droughts, hurricanes), and the impacts of these climate events on food security initiatives. Additionally, it captures the current food security status and outlines any recommendations made by the studies or identifies areas needing further research. This structured approach ensures a comprehensive analysis of the interactions between climate change and food security as informed by the included literature.

## 3. Results and Discussion

### 3.1. Characteristics of Selected Articles

The nine studies included in this scoping review share several defining characteristics that shape both the strengths and limitations of the evidence base. Most employ qualitative or mixed-methods designs, combining household surveys, participatory rural appraisal techniques, and in-depth interviews to document lived experiences of climate stress, food insecurity, and mobility decisions [21,22,23,24,25]. Among these, one study specifically examined the experiences of children living in refugee camps [26], whereas the others focused on adult populations. The demographic analysis indicated a predominance of male climate refugees, although there has been increasing participation by women in several countries [25]. Temporally, most studies rely on cross-sectional or retrospective data, often using perceived changes in rainfall patterns and self-reported food security experiences rather than longitudinal or experimental designs [24,25]. Thematically, internal migration dominates the evidence base, while forced displacement and refugee contexts are less directly examined, appearing primarily in studies of internally displaced populations or rural–urban migrants [26,27]. Food security is most commonly operationalized through production- and access-oriented indicators, such as crop yields, food shortages, coping strategies, and market access, with comparatively limited attention to dietary quality, nutritional outcomes, or destination food environments [27,28]. Regarding food security initiatives, the most common strategies identified were distribution of food aid and community support, including loans and food sharing among community members [26]. Collectively, these characteristics indicate a literature that is strong in contextual vulnerability assessment but more limited in standardized measurement, longitudinal analysis, and evaluation of food security initiatives across different stages of mobility.

**Table 1:** Overview of Included Studies.

Author(s) and Year of Publication	Study Aim	Study Design	Study Location	Study Population	Climate Change Events (Drought, Rain, Etc.)	Impact of Climate Change on Food Security Status	Food Security Initiatives	Identified Recommendations
Afifi, T. et al. (2014) [21]	To explore the interrelations between rainfall variability, food insecurity, and human mobility within three distinct villages in Same-Kilimanjaro, Tanzania.	A mixed-method approach combining expert interviews, a household survey of 165 households, and Participatory Research Approach (PRA) sessions with community members.	Same-Kilimanjaro, Tanzania.	Local communities within three villages: Vudee, Bangalala, and Ruvu Mferejini, each differing in elevation and precipitation levels.	Rainfall variability	Changes in rainfall patterns, including unpredictable droughts and inconsistent seasonal rains, have led to decreased agricultural productivity.	**Ndivas (water ponds):** These traditional water storage facilities play a crucial role in irrigation and maintaining water supply during dry periods. **Diversification of crops and income:** Some communities have begun diversifying their crops and seeking alternative income sources to reduce their reliance on traditional rain-fed agriculture.	**Maintenance and expansion of Ndivas**: Enhancing the capacity and maintenance of traditional water storage to better support agricultural activities. **Community support and remittances**: Leveraging remittances and community support systems to improve local resilience and food security. **Policy support**: The study recommends that policies focus on supporting sustainable agricultural practices and improving water management to mitigate the impacts of rainfall variability.
Milan et al. (2014) [22]	To provide insights into how four mountain communities in Guatemala are experiencing and responding to the challenges posed by rainfall variability.	A multi-method approach, combining household surveys, expert interviews, and Participatory Research Approach (PRA) sessions.	Cabricán region, Quetzaltenango, Guatemala.	Rural mountain communities in the Western Highlands of Guatemala, predominantly of the Mam ethnic group.	Rainfall variability	Rainfall variability has led to increased food insecurity in the region, primarily due to its impact on the agricultural output of the rain-fed milpa system, which is a cornerstone of local subsistence farming.	**In-situ diversification and use of resilient crop varieties**: Farmers have adapted by diversifying their crops and using varieties more tolerant to the changing climate. **Water conservation measures**: Implementation of water reservoirs to collect rain during the rainy season, enhancing water availability for agriculture.	Enhancing support for local adaptation strategies, such as improving agricultural practices and diversification beyond traditional farming to include non-agricultural income sources.
Murali & Afifi (2014) [23]	To investigate the impacts of rainfall variability on agriculture, food security, livelihoods, and human mobility in the Janjgir-Champa district of Chhattisgarh, India.	A combination of qualitative (expert interviews and Participatory Research Approach sessions) and quantitative methods (a household survey involving 180 households).	Janjgir-Champa district, Chhattisgarh state, India.	Farmers and farm laborers in four villages within the Janjgir-Champa district.	Rainfall variability	Rainfall variability has significantly impacted agricultural productivity in the region, leading to crop failures and increased food insecurity.	**Adoption of short or medium-duration rice varieties**: To cope with reduced rainfall during crucial growth periods. **Utilization of the Bongo Reservoir for irrigation**: Ensures water availability during the monsoon and potentially for winter crops.	Enhancing local disaster policies, improving spatial planning, building codes, infrastructure practices, and propagating updated flood information through advanced warning systems.
Rademacher-Schulz et al. (2014) [24]	To investigate the shifting patterns of seasonal migration in Northern Ghana in response to rainfall variability and food insecurity.	A mixed-methods approach, including a household survey of 158 households, participatory rural approaches (PRA), and expert interviews.	Nadowli District, Upper West Region, Ghana.	Households in rural Savannah communities in the Nadowli District of the Upper West Region of Ghana.	Rainfall variability	Rainfall variability leads to decreased crop yields and increased food insecurity.	N/A	Differentiating between coping and adaptation strategies, emphasizing the need to enhance responsive adaptation that can mitigate the adverse effects of rainfall variability on food security.
Warner and Afifi (2014) [25]	To explore under what circumstances households use migration as a risk management strategy when facing rainfall variability and food insecurity, particularly in the context of climate change.	A mixed-methods approach, including household surveys and participatory research across several districts in eight countries.	Guatemala, Peru, Ghana, Tanzania, Bangladesh, India, Thailand, and Vietnam.	Households in rural communities dependent on rain-fed agriculture.	Rainfall variability	Rainfall variability negatively impacts agricultural outputs, leading to increased food insecurity for households that depend on agriculture for their livelihoods.	N/A	Emphasizing the need for policies that recognize migration as a legitimate adaptation strategy and support vulnerable households in enhancing their resilience.
Jacobson et al. (2019) [28]	To explore the relationships between climate change, migration, and food security in Northwestern Cambodia.	A case study approach, integrating climate data analysis with surveys of households affected by migration.	Northwest region of Cambodia	Households in three communities around the Tonle Sap Lake, which are differentially affected by climate change and have varying migration patterns.	Rainfall variability and the occurrence of extreme events like droughts	Climate variability, particularly in rainfall, leads to reduced agricultural yields and increased food insecurity.	**Livelihood adaptations**: Livelihood adaptations can help communities cope with the changing climate without relying solely on migration.	Enhancing local adaptation strategies that address both the direct impacts of climate change and the socio-economic factors exacerbated by migration. Improving agricultural practices and diversifying income sources to reduce the reliance on climate-sensitive agriculture.
Ahmad & Afzal (2020) [29]	To assess the types of flood mitigation measures adopted and the adaptive capacities of flood-affected households in the Punjab province of Pakistan.	A cross-sectional design with data collected from 840 flood-affected households.	Punjab, Pakistan	Households affected by floods in the Punjab province, particularly those in the districts of Rahim Yar Khan, Muzaffargarh, and Rajanpur.	Flood events	The implications for food security are indirectly addressed through the discussion on economic losses and disruptions caused by floods, affecting agricultural productivity and household livelihoods.	**Flood mitigation measures**: Flood mitigation measures indirectly contribute to stabilizing household food security by protecting assets and enabling economic recovery post-flood.	Enhancing local disaster policies, improving spatial planning, building codes, and infrastructure practices, and propagating updated flood information through advanced warning systems.
Schwerdtle et al. (2021) [27]	To explore the health and mobility challenges faced by migrants from rural Bhola to urban Dhaka, Bangladesh, in the context of climate change.	Qualitative study utilizing semi-structured in-depth interviews.	Dhaka, Bangladesh	Migrants who have moved from rural Bhola to an urban slum in Dhaka, Bangladesh.	Climate change in general	N/A	N/A	Developing policies that integrate a migrant-centric perspective, which includes recognizing the various health risks and the “risk exchange” migrants undergo.
Kemei, et al. (2023) [26]	To examine the insecurities experienced by internally displaced persons (IDPs), particularly children, in the Burayu camp in Ethiopia.	An exploratory qualitative case study utilizing an intersectionality theoretical lens to investigate the perceived forms of insecurities among IDPs.	Burayu camp, located near Addis Ababa in Ethiopia.	20 children, 20 parents or guardians, and 13 service providers within the IDP community.	Climate change in general	The study indirectly touches on the impact of climate change through the challenges of food insecurity exacerbated by displacement.	**Humanitarian food aid:** Humanitarian food aid is a primary initiative addressing food insecurity among IDPs.	Enhancing the effectiveness of food distribution programs and integrating efforts with broader community support initiatives to improve the food security status of IDPs.

### 3.2. Mapping Evidence Gaps

Across the nine included studies, the evidence base clusters strongly around one dominant pathway. Climate and environmental stressors, especially rainfall variability, drought, flooding, and seasonality shifts, undermine agricultural production and income, which then drive food insecurity and trigger mobility as a risk management response. This pattern is most explicit in Warner & Afifi (2014) study [25] and related site-specific analyses in Tanzania, Guatemala, India, and Ghana, where food insecurity is repeatedly positioned as the key mediating mechanism linking climate stress to migration decisions [21,22,23,24,25]. Even when the focal hazard differs, such as floods in Pakistan, the implied chain is similar. Climate shocks damage livelihoods, reduce access to food, and increase the likelihood of mobility, or of conditions that can precipitate displacement [29]. This consistency is a strength of the literature. It also reveals a limitation. The field often reaffirms the same causal storyline while offering fewer concrete insights into which food security initiatives measurably prevent displacement, or improve food security outcomes after mobility occurs.

A second clear finding, but one the literature handles unevenly, is that mobility is not reliably “adaptive.” As detailed in Section 3.6, migration can be maladaptive, with food insecurity and debt persisting or worsening, and with social costs that may erode longer-term resilience [28]. The multi-country synthesis similarly distinguishes “content” migration from “erosive” migration, and highlights “trapped populations” who are highly vulnerable yet unable to migrate [25]. Yet across studies, these categories are not applied consistently, and the criteria for success vary. Some studies treat remittances or temporary consumption smoothing as a marker of success, while others emphasize longer-term livelihood resilience, social wellbeing, or the capacity to maintain food production. This definitional drift makes it difficult to align results across settings, and it encourages contradictory conclusions that are partly artifacts of measurement choice rather than true empirical disagreement.

Definitions and outcomes also shift from study to study in ways that weaken comparability. “Food security” is frequently proxied by crop yields, self-reported food shortages, or coping behaviors in the rainfall–migration studies [21,22,23,24,25]. In contrast, the qualitative study by Schwerdtle et al. (2021) frames food security through affordability, food quality and safety, dietary change, and the loss of home food production after rural–urban migration, showing “improvements” and “declines” can coexist depending on which dimension is measured [27]. The Ethiopia internally displaced persons (IDPs) study by Kemei et al. (2023) positions food insecurity within a broader insecurity ecology in camps, where rations, market access, and competing needs shape hunger and malnutrition risks [26]. This is closer to a humanitarian framing than an agricultural livelihoods framing [26]. The result is that findings do not always line up because the underlying constructs are not stable. Many studies also blur migration, displacement, and refugeehood. Most of the included articles focus on internal migration. Only a subset directly engages displacement-like conditions, and even then, the legal and operational distinctions are often not made explicit [26,27].

The largest gap, relative to the review question, is that the evidence base is thin on food security initiatives as interventions with evaluated outcomes. Several studies recommend safety nets, seasonality-sensitive food aid, livelihood diversification, or local adaptation investments, but they rarely test these strategies or compare initiative types with measurable effects on food security status among migrants, displaced people, or refugees [24,25]. The Pakistan flood study [29] focuses on household mitigation strategies rather than food security programming, and the Bangladesh [27] and Ethiopia [26] studies highlight downstream vulnerabilities at destinations without isolating which program designs reduce food insecurity in practice. In short, the literature is stronger at diagnosing the climate–food insecurity–mobility relationship than at identifying effective food security initiatives before displacement, during mobility, or after arrival.

**Figure 2:**
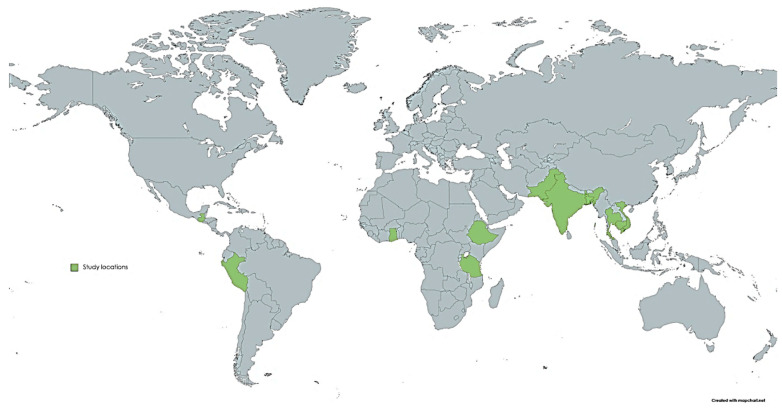
Geographic Locations of Included Studies (created by https://www.mapchart.net/).

One further limitation of the mapped evidence is its narrow geographic scope. All nine studies were conducted in the Global South, predominantly in low- and middle-income, agrarian, or informally urbanizing contexts in Africa, Asia, and Latin America (Figure 2). While these settings are appropriately prioritized given their high exposure to climate stress and food insecurity, this concentration limits the generalizability of findings across different political, institutional, and food system contexts. The absence of studies from high-income or mixed migration settings constrains understanding of how climate-related food insecurity and displacement may unfold under stronger social protection systems, different labour markets, or more formalized food security initiatives, and highlights a clear gap for future comparative research. Together, these geographic and contextual limitations highlight the importance of examining how climate change impacts on food security and mobility vary across places.

### 3.3. Geographic Disparities in Climate Change Impact

Climate change does not impact all regions of the world equally [30]. Geographic, economic, and infrastructural factors combine to create significant disparities in how climate change affects different parts of the globe, particularly concerning food security [31].

#### 3.3.1. Variation in Climate Vulnerability

Susceptibility to climate impacts varies widely, influenced by factors such as geographic location, topography, and existing climatic conditions [32,33,34,35]. For instance, island nations and coastal regions are disproportionately affected by sea-level rise and hurricanes [32,33], while arid and semi-arid regions face heightened risks from droughts and heatwaves [34,35]. These geographic specificities shape the types of climate threats communities face and determine the specific local actions that are needed to reduce negative impacts. For example, in South Asia, particularly in countries like India, Bangladesh, and Pakistan, the primary climate threats include flooding, cyclones, and sea-level rise [36,37]. These events disrupt agricultural cycles and directly threaten food production systems, especially in coastal areas where saline intrusion into freshwater systems poses a significant threat to rice cultivation [38]. For instance, between 1962 and 1988, Bangladesh lost about 4% of its total rice production each year due to floods, which equated to approximately 0.47 million tonnes annually. This loss accounted for nearly 30% of the country’s average annual food grain imports, which stood at around 1.6 million tonnes [39]. The dense population and high rates of rural poverty make these communities more vulnerable [36] and force many households to choose between migrating within the country or enduring harsh conditions where they are. In 2022, the Bay of Bengal Cyclone Sitrang demonstrated the destructive power of such climate events, killing 35 people, displacing over 20,000, and causing more than $35 million in damages across Bangladesh [40].

Conversely, Sub-Saharan Africa, which is heavily dependent on rain-fed agriculture, experiences severe food security challenges due to increased frequency and intensity of droughts, which not only decrease crop yields but also degrade soil quality [41,42]. For example, countries like Ethiopia and Sudan have experienced recurring droughts that lead not only to crop failure but also to severe water shortages, which exacerbate food insecurity and malnutrition [43,44]. The socioeconomic conditions, including widespread poverty and limited access to technology, magnify the impact of these climatic changes, pushing communities to rely heavily on humanitarian aid and migration as coping strategies [45,46,47]. Furthermore, regions such as the Dry Corridor in Central America, which includes parts of Guatemala, Honduras, and Nicaragua, are experiencing increased drought frequency and intensity [48]. Similarly, in parts of Brazil, variability in rain patterns has led to significant disruptions in both subsistence and commercial agriculture [49].

#### 3.3.2. Economic and Infrastructure Disparities

Regions with limited economic resources and inadequate infrastructure are less able to adapt to climate change, which makes them more vulnerable to its impacts [50,51]. Economic disparities are evident when comparing the adaptive capacities of developed and developing nations [50,51]. Developed countries are generally considered better equipped to handle the impacts of climate change due to their access to superior resources: financial, technological, and social [51]. They often have the financial means to invest in advanced agricultural technologies [52], comprehensive flood defenses [53], and robust public health systems [54], which all are critical for mitigating and adapting to climate impacts. In contrast, developing countries, with their constrained budgets and often fragile infrastructures, often struggle to implement extensive climate adaptation or mitigation strategies [50,51].

#### 3.3.3. Socio-Economic Factors

The impact of climate change is also mediated by socio-economic factors that influence vulnerability and adaptive capacity [55,56,57,58]. Communities with higher poverty rates, less education, and limited access to services are particularly vulnerable to climate disruptions [56,57,58]. These factors exacerbate geographic inequalities, as impoverished rural regions often bear the worst of climate impacts while lacking the resources needed to adapt effectively [55,58,59]. This disparity is evident in the contrast between urban and rural responses to climate threats [59]. Urban areas, with better infrastructure, often have more resources and strategies for adaptation. In contrast, rural areas, particularly in less developed countries, lack such support systems and infrastructure, which leads to more significant disruptions in their food security and overall livelihoods [59]. The geographic disparities in climate change impacts require customized responses that consider local conditions, resources, and vulnerabilities [30]. Effective adaptation and mitigation strategies must be locally informed and globally supported, acknowledging the diverse challenges faced by different regions.

### 3.4. Patterns of Movement and Migration in Response to Environmental Changes

Migration in response to environmental changes, including climatic events, reflects a complex mix of socio-economic factors, geographical distances, and individual or community resource availability [60,61,62,63]. These patterns that are shaped by various determinants require comprehensive policy frameworks to address the diverse needs of migrants and the communities affected by migration.

**Internal vs. International Migration:** The vast majority of migrations are internal, with individuals relocating within their own countries rather than crossing international borders [60,61,64,65]. This trend is exemplified by massive internal migrations in countries like China, where rural-to-urban migration predominates as people seek better economic opportunities [66]. International migration, while significant, involves a smaller proportion of the global migrant population and is often influenced by stricter barriers such as immigration policies and higher relocation costs [64,65,67,68].

**Proximity and Social Networks:** Migration tends to occur over shorter distances and is heavily influenced by social networks [60,62,69]. Migrants often move to locations where they have family or community connections, which can provide immediate support and resources upon arrival [60,65,70]. This pattern is evident in disaster response situations, where displaced people usually seek shelter with friends or relatives rather than moving to unfamiliar places.

**Rural-to-Urban Migration:** A common pattern within internal migration is the movement from rural to urban areas [45,71,72]. This migration trend, which is often driven by the search for employment and better living conditions, is also a step towards more distant migrations as individuals transition through various stages of economic stability [60,71].

**Temporary vs. Permanent Migration:** Migration in response to environmental factors can be temporary or permanent [60,61,63,73]. Displacements that are caused by sudden environmental disasters, such as floods and hurricanes, are typically temporary [62,63,74]. Affected individuals often return to their original locations to rebuild, which indicate a strong attachment to place and community [74,75,76]. Permanent migration may result from prolonged environmental deterioration that leaves areas uninhabitable or economically unviable [62,63]. The movement patterns of migrants are dictated by a combination of environmental, economic, and social factors [60,61,62,63]. Policies designed to support migrants must therefore consider these diverse influences to effectively integrate migrants into new communities and ensure the sustainability of both origin and destination areas. As environmental impacts on migration continue to evolve, adaptive policy responses will be important in managing the complexities of migration and meeting the needs of affected populations.

### 3.5. Climate Variability and Food Security Challenges for Displaced Populations

The status of food security among climate refugees is intricately linked to the variability of climatic conditions, particularly rainfall. For instance, as detailed in the study from Same-Kilimanjaro, Tanzania, significant rainfall shortages resulted in increased food insecurity and forced many individuals to migrate in search of better living conditions [21]. This migration is not merely a relocation but a survival strategy in response to the inability to maintain a stable food supply [21]. The socio-economic characteristics of these communities further compound the challenges. The research showed that while wealthier households are more likely to migrate in order to mitigate the risks of food insecurity, poorer households often remain and face higher risks of food shortages [21]. This disparity highlights the need for targeted interventions that address the specific vulnerabilities of different groups within climate-impacted populations. The food security status of the communities in Cabricán, Guatemala, is critically tied to rainfall patterns due to their dependence on subsistence agriculture [22]. Milan et al. (2014) reported that changes in rainfall over the past two decades have led to increased instances of crop failure, which significantly impacted food availability and exacerbated food insecurity [22]. This study noted that nearly 78% of households have experienced food scarcity in the last decade. With agriculture being the major source of livelihood, any shift in rainfall patterns directly affects food production, leading to periods of intense food insecurity. This situation is further strained by limited local market access and a lack of diversity in available food products, which affects the nutritional intake of the community. The persistent threat of becoming “trapped” without sustainable livelihood or migration options highlights the precarious nature of food security in this region [22]. Similarly, the food security status of the population in the Janjgir-Champa district of India is critically dependent on the monsoon season [23]. Since agriculture is the primary livelihood, any deviation in expected rainfall patterns directly affects food availability. The report by Murali & Afifi (2014) documented a growing trend of shorter and less predictable rainy seasons, which significantly diminishes agricultural yields and increases food insecurity [23]. This insecurity is further exacerbated by the limited success of government programs like the Mahatma Gandhi National Rural Employment Guarantee Act, which aims to provide secure employment but has not sufficiently mitigated the challenges posed by rainfall variability. As a result, many residents resort to migration as a coping strategy, seeking employment in more urbanized areas where food availability is less dependent on local agricultural output [23]. The food security status among climate refugees in Punjab, as detailed by Ahmad et al. (2020), is linked to the frequency and intensity of flood events, which regularly impact the region [29]. The study identified significant socioeconomic determinants that influence the adoption of flood mitigation strategies, which in turn affect food security. Households with better financial resources or those headed by individuals with higher educational levels tended to adopt more effective flood mitigation measures, thereby enhancing their food security. Conversely, less affluent or less educated families faced greater challenges in securing their food needs during crises [29]. The food security status of climate refugees is thus a complex interaction of environmental hardships, socio-economic factors, and the effectiveness of local and national food security initiatives, which will be discussed in the following sections. As climate change continues to pose a significant threat to stable food systems, understanding the specific needs and responses of climate refugees becomes crucial in formulating effective policies and programs that ensure their food security and overall well-being. 

### 3.6. The Dual Impact of Climate-Induced Migration on Food Security 

As climate change continues to impact global weather patterns, particularly rainfall variability, its effects on food security and migration are becoming increasingly complex and interlinked. This collection of studies has explored how different forms of migration serve as mechanisms for households to manage food security risks. The study by Warner and Afifi (2014) provided insights from multiple countries on how households employ migration as a risk management strategy in response to rainfall variability and associated food insecurities [25]. It identified two main types of migration: ‘content’ and ‘erosive’. Content migration, associated with resilient households, involves using migration as an effective adaptation strategy. These households typically engage in migration to improve livelihood opportunities through remittances and knowledge transfer, thus enhancing their food security. Such migration often involves moving to areas with more stable climatic conditions and better economic opportunities, enabling these households to implement and benefit from enhanced agricultural techniques and diversified income sources [25]. Conversely, erosive migration, which is prevalent among more vulnerable households, often results in degraded living conditions and increased food insecurity. These households who are forced to migrate due to severe climatic disruptions and the failure of local adaptation measures frequently find themselves in situations where migration exacerbates their vulnerability. For instance, they might migrate to urban areas where they face high living costs, poor job prospects, and limited access to healthy food, further deepening their food insecurity. This type of migration is typically a last resort and highlights the challenges in managing the risk of rainfall variability without adequate support systems or successful adaptation strategies in place [25]. The authors emphasized the need for targeted interventions to prevent migration from becoming an erosive process and suggested that strengthening local resilience and support structures could mitigate the need for such desperate measures [25]. A subsequent study by the same group of authors identified four distinct migration patterns that arise in response to environmental changes impacting livelihoods and food security [77]: (1) Resilient Migration: Households proactively use migration to enhance resilience and access better economic opportunities, benefiting from diverse livelihood options; (2) Migration for Survival: Migration acts as a necessity for households facing immediate threats like rainfall variability, which acts to provide temporary relief but not long-term resilience; (3) Erosive Migration: For the most vulnerable, migration is a desperate act that worsens their situation and often lead to debt and social instability; and (4) Trapped Populations: Some households who lack resources to migrate endure severe impacts from environmental changes and face significant food and livelihood insecurity [77]. These patterns highlight the need for policies that address the varied impacts of climate change on different groups and enhance support and resilience.

The research by Jacobson et al. (2019) highlighted the complex relationship between climate-induced migration and food security status among climate refugees in Cambodia [28]. Migration, while often a response to immediate food insecurity caused by crop failures and other climate-related adversities, does not necessarily lead to improved food security. In many cases, migration results in labour shortages that further exacerbate food insecurity in the originating communities. Additionally, the remittances sent back by refugees often fail to compensate for the loss of agricultural labour, leading to a cycle of food insecurity and economic hardship that can persist across seasons and years. The study suggested that migration can become a maladaptive response if not accompanied by effective strategies that address both the causes and consequences of food insecurity in a changing climate [28]. The study by Rademacher-Schulz et al. (2014) presented a complex picture of how changes in seasonal migration patterns affect food security among climate refugees [24]. Traditionally, migration during the dry season helped manage food resources by decreasing local dependence and increasing income through remittances. However, an emerging shift toward migration during the rainy season, when agricultural labour is most needed, suggests a distress-driven migration pattern, potentially indicative of severe food insecurity. This shift may be driven by immediate household food needs, predicting a poor harvest or as a reactive measure to an already failing crop yield due to erratic rainfall. Such migration, while providing short-term relief through remittances, may undermine long-term food security by depleting local labour needed for farming, thereby further exacerbating the cycle of food insecurity and increasing household vulnerability [24]. According to Kemei et al. (2023), the food security status among IDPs in Ethiopia is dire, with frequent food shortages leading to malnutrition and increased vulnerability to diseases [26]. These conditions are exacerbated by the IDPs’ reliance on humanitarian food aid, which is often insufficient to meet the nutritional needs of the population. The study highlighted that food insecurity not only affects the physical health of the displaced individuals but also their psychological well-being, as constant worry about food availability adds to the stress and trauma of displacement. Moreover, the lack of adequate food exacerbates the challenges faced by IDPs in achieving economic stability and integrating into new communities, thereby maintaining a cycle of poverty and insecurity [26]. Further, the findings from Schwerdtle et al. (2021) indicated a complex impact on the food security status of climate refugees, characterized by both gains and losses [27]. In their new, and often urban, environments, refugees face difficulties like inadequate shelter and sanitation, which indirectly affect their ability to store food safely and maintain a nutritional diet. Additionally, the urban food environment presents a double-edged sword: while refugees have more frequent access to markets than in rural areas, the affordability and quality of food can be significantly lower. The study elucidated the critical intersection of health, mobility, and food security, emphasizing that while migration can alleviate some immediate climate-related food insecurities, it introduces new challenges in urban settings, where refugees must navigate complex and often expensive food systems [27]. The findings highlighted the urgent need for comprehensive, context-sensitive strategies that not only address immediate food shortages but also aim to enhance long-term resilience and adaptation capabilities. This involves strengthening local agricultural practices, improving urban food systems, and supporting refugees through better integration policies and support networks. Future research needs more consistent definitions and harmonized indicators, especially for food security and for mobility categories, so that “adaptive versus maladaptive” claims become comparable across contexts.

### 3.7. Initiatives to Address Food Security and Support Displaced Populations Amidst Climate Challenges

In examining the impacts of climate change on displaced populations, several studies have highlighted the significant challenges and adaptations concerning food security, emphasizing the crucial roles of community-based support and humanitarian aid [26,27]. Kemei et al. (2023) explored the severe conditions faced by IDPs in Ethiopia by focusing on the food insecurity that affects these vulnerable populations [26]. One of the primary initiatives to combat food insecurity in Ethiopia involves humanitarian aid, which plays a key role in providing food relief to IDP camps. However, the study indicated that these efforts often face challenges such as inconsistency in food supplies and the limited capacity to meet the nutritional needs of the growing IDP population. The research suggested that enhancing the effectiveness of food distribution programs and integrating these efforts with broader community support initiatives could improve the food security status of IDPs, thereby mitigating the adverse effects of displacement on children and their families [26]. Schwerdtle et al. (2021) explored the complex migration dynamics resulting from climate-induced relocations in Bangladesh [27]. The study revealed that refugees migrating from rural areas to urban centers, particularly Dhaka, experience a ‘risk exchange,’ where certain climate-related risks are replaced by new urban vulnerabilities. This includes a shift in food security risk, where refugees escape immediate threats like crop failure due to saline intrusion and flooding but encounter new challenges in urban settings, such as higher food prices and reduced access to nutritious and fresh foods. The paper highlighted the vital role of community-based support and social networks that help mitigate some of these new food security risks by facilitating access to shared resources and information about food and job opportunities in the city [27]. 

Furthermore, by examining diverse geographic regions and the specific challenges they face due to climate change, several studies highlighted the innovative food security initiatives being implemented across the globe. In response to climate-induced challenges such as erratic rainfall and resulting food insecurity, communities in Same-Kilimanjaro have implemented various initiatives aimed at improving food security [21]. The research by Afifi et al. (2014) illustrated how traditional water conservation methods, specifically Ndivas (local water ponds), play a key role in sustaining agricultural activities during periods of reduced rainfall [21]. These initiatives are critical in regions where agriculture is predominantly rain-fed and where even slight shifts in rainfall patterns can precipitate significant food shortages. Moreover, the study highlighted the importance of diversified livelihood strategies as a buffer against climatic shocks. By engaging in alternative economic activities, communities can reduce their sole dependence on agriculture, thereby enhancing their resilience to climatic variabilities. These initiatives are particularly vital for climate refugees who are forced to migrate due to deteriorating environmental conditions in their home areas. Enhancing and scaling up such adaptive strategies can be effective in improving food security among climate refugees, offering more stable and sustainable solutions even in the face of ongoing climate adversity [21]. The study conducted by Murali & Afifi (2014) in the Janjgir-Champa district of Chhattisgarh, India, examined the distressing impacts of rainfall variability on agriculture and food security [23]. It highlighted several initiatives aimed at adapting to these changes and enhancing food security among climate refugees. These initiatives included adopting short or medium-duration rice varieties to cope with reduced rainfall during crucial growth periods. Additionally, the use of communal resources like the Bongo Reservoir for irrigation during the monsoon reflected a strategic attempt to stabilize agricultural output in the face of erratic rainfall patterns. This strategic resource allocation was key in sustaining agricultural productivity and thus food security in an area predominantly dependent on monsoon-fed agriculture [23]. The research by Ahmad et al. (2020) explored the household-level flood mitigation strategies and adaptive capacities in flood-prone areas of Punjab, Pakistan, revealing several initiatives aimed at enhancing food security among climate refugees [29]. These strategies included the construction of houses with reinforced materials and the elevation of ground floors to prevent floodwaters from entering homes, thereby securing food storage areas. Additionally, households engaged in precautionary saving to buffer against potential economic shocks caused by flooding. These measures were integral to maintaining food availability and security during flood events, demonstrating an active engagement by communities in strengthening their resilience against climate-induced food insecurity [29]. Jacobson et al. (2019) provided an in-depth analysis of the consequences of climate-driven migration in North-western Cambodia, which revealed the importance of targeted food security initiatives for climate refugees [28]. The study highlighted the necessity of adopting livelihood adaptations that directly address the demographic shifts caused by the migration of predominantly young males from agricultural areas. These adaptations included enhancing the resilience of agricultural practices through improved irrigation systems and diversified cropping strategies to reduce dependency on traditional rain-fed agriculture. The initiatives aimed to mitigate the labour shortages and gender role implications that arise from migration, ensuring that the agricultural productivity remains sufficient to support food security in the affected regions [28]. Milan et al. (2014) examined the effects of rainfall variability on food security and migration in Cabricán, Guatemala [22]. This study highlighted how the local communities who are reliant on rain-fed agriculture have adopted several in situ adaptation strategies to combat increasing climatic unpredictability. One such initiative involved crop diversification, where farmers began planting different varieties of maize, beans, and other staple crops that are more resilient to climatic extremes such as drought and heavy rainfall. Additionally, the introduction of drought-resistant crop varieties and the use of water reservoirs to collect rain during the rainy season were practices aimed at sustaining agricultural output despite fluctuating rainfall. These efforts were important for maintaining food security in a region where agriculture is the lifeline of the community [22]. In summary, the comprehensive research from diverse global regions underlined the critical need for innovative and adaptive strategies to bolster food security in the face of climate change. From the water conservation techniques in Kilimanjaro to the strategic crop diversification in Guatemala, these studies collectively demonstrated the resilience and adaptability of communities under climatic stress.

Amidst the ongoing challenges posed by climate variability, several studies provided critical insights into the dynamics of migration as a strategy for managing climatic risks and enhancing food security [24,25]. Warner and Afifi (2014) presented valuable data showing that while migration is often viewed as a response to climatic stressors, its effectiveness varies greatly depending on the vulnerability and resilience of individual households [25]. The study highlighted the importance of designing food security initiatives that are adaptable to the diverse needs of households affected by climate variability. Initiatives must focus on both enhancing the adaptive capacities at the local level and providing support for refugees and their families. This includes improving access to education, financial services, and sustainable farming practices that are less dependent on consistent rainfall patterns. By recognizing the types of migration (content vs. erosive), initiatives can be tailored to strengthen household resilience against climatic shocks, rather than exacerbating existing vulnerabilities [25]. Rademacher-Schulz et al. (2014) detailed how seasonal migration from Northern Ghana, particularly during the dry season, has historically functioned as a dual adaptation and coping mechanism in response to food insecurity and environmental changes [24]. This migration typically involves moving to the southern regions of Ghana, where agricultural opportunities are more robust due to better climatic conditions. These movements are strategic; refugees engage in agricultural labour or other income-generating activities in the south, sending remittances back home that bolster household food security. Additionally, this migration reduces pressure on local resources during the dry season, which temporarily alleviates food scarcity back in their communities [24]. In conclusion, the diverse range of initiatives documented across various studies underscores the critical importance of adopting flexible and innovative strategies to enhance food security amid climate challenges and displacement. These initiatives, ranging from community-based support and humanitarian aid to strategic adaptations in agricultural practices and infrastructure, highlight a proactive approach in responding to the immediate and long-term needs of displaced populations. However, mapping the current evidence suggest that studies need designs that can evaluate initiatives or interventions, not just describe risk pathways. For example, comparative evaluations of seasonality-sensitive safety nets, community-based livelihood supports, cash or food assistance in displacement settings, and urban food access programs for climate migrants.

### 3.8. Effectiveness and Challenges of Migration as an Adaptation Strategy to Support Food Security

Migration, when approached as a strategic response rather than a forced displacement, has emerged as a key adaptive strategy to environmental changes and climate impacts [60,78,79]. Migration allows individuals to relocate from high-risk areas to more secure environments, thus diversifying and mitigating risk. This strategy is particularly vital in regions where local adaptation options are limited or insufficient [80,81]. By moving away from areas of environmental decline or severe weather patterns, migrants can reduce their vulnerability and enhance their overall security [80,81]. Moreover, the financial remittances sent back by migrants play a key role in stabilizing the economies of origin communities [60,78,82]. These funds can help stabilize households during adverse environmental conditions by smoothing consumption and maintaining access to essential goods and services [82,83,84]. For example, studies have shown that remittances can exceed the volume of official development assistance and provide a more reliable capital flow than international aid [83,85]. Further, migration facilitates the transfer of skills, knowledge, and cultural practices which can contribute to the social and economic development of the origin communities [78,79]. Migrants often return with new perspectives and skills, which can be utilized to enrich their communities by introducing innovative practices and technologies [60,78]. This transfer is not just limited to economic benefits but also includes enhancing social networks and expanding the social capital of communities, which is vital for broader community resilience [60,78]. Finally, migration can alleviate pressure on local resources by redistributing population density. This not only reduces per capita demands on local resources but also helps manage environmental degradation in areas experiencing overpopulation [79].

Not all migration outcomes are positive. In situations where migration is forced or driven by desperation, it can lead to “erosive” outcomes where migrants end up in worse conditions, such as poor employment opportunities, inadequate living conditions, and social exclusion [25,86]. Moreover, rapid urban migration can overburden infrastructure, particularly in cities that are unprepared for large population influxes. This can exacerbate urban poverty and create new environmental stresses in areas that may already be vulnerable to climate impacts [87,88]. Additionally, migrants often face disparities in socio-economic status compared to local populations, which can limit their ability to secure employment and access to adequate housing and services [89]. Finally, while migration can relieve pressure on local resources, it can also result in a ‘brain drain,’ where the departure of skilled and able-bodied individuals leaves behind a gap in the local workforce. This gap can limit local development and adaptation efforts, particularly in rural and underdeveloped areas [6,90].

The dual nature of migration as both a potential benefit and a risk highlights the need for policies and programs that maximize its adaptive benefits while minimizing its risks. For example, policies that enhance integration by facilitating migrant access to the labour market and ensuring the provision of basic services such as healthcare and education help migrants stabilize quickly and contribute positively to their new communities [91]. Another strategic approach involves forming migrant networks that can collectively pool resources to invest in community development initiatives, such as healthcare, education, and climate-resilient infrastructure [92]. Moreover, developing support programs that enable the transfer of knowledge and skills between migrants and their communities of origin [93,94]. This can be achieved through diaspora networks that invest in local infrastructure, such as schools and healthcare facilities, and enhance the adaptive capacities of these communities [95]. Further, it is important to ensure that migrants have secure legal status and access to rights that allow them to live and work without fear of exploitation or discrimination [92]. This encourages the circular movement of migrants, which supports the transfer of funds and knowledge back to their communities of origin. Taken together, these measures can maximize the benefits of migration as an adaptive strategy while reducing its associated risks.

### 3.9. Alternative Strategies for Food Security Initiatives

As climate change continues to threaten food security globally, particularly for climate refugees and displaced populations, moving toward alternative strategies that go beyond conventional agricultural practices are critical. These strategies must be robust, adaptable, and scalable to address the unique challenges faced by climate-impacted communities effectively. Community-based agroecological projects offer a sustainable alternative to traditional agriculture with a specific focus on biodiversity, ecological harmony, and local resilience [96]. These systems can be particularly effective in areas where climate variability impacts traditional farming practices. By promoting a diversity of crops and using locally adapted seeds, communities can better withstand climatic extremes and reduce dependency on single-crop economies [97]. Additionally, agroecology enhances soil conservation and water management, which are critical in maintaining crop yields in changing climatic conditions [98]. Another potential strategy is the use of climate-smart agriculture (CSA), which includes practices and technologies that sustainably increase productivity, enhance resilience to climate change, and reduce or remove greenhouse gas emissions where possible [99,100]. CSA practices such as conservation agriculture, improved crop varieties, and efficient water use can be particularly effective in regions experiencing adverse climatic changes [99,100]. However, it is important to note that CSA interventions require substantial growth in affected communities’ capabilities in terms of qualified personnel, technology, and financial resources [101]. Therefore, training and supporting climate refugees to implement CSA practices can help mitigate the impacts of climate change on their food security.

For refugees resettled in urban areas, space-efficient agricultural techniques such as vertical farming can provide a viable means of producing food [102,103]. These systems utilize small areas to grow crops in vertically stacked layers, often incorporating hydroponic or aquaponic systems that reduce water usage and eliminate the need for soil [102,103]. Urban agriculture not only helps in providing fresh produce to urban dwellers but also creates green spaces that can improve the urban microclimate and overall well-being of the community. Moreover, Integrated Food Energy Systems (IFES) offer a holistic approach to sustainability by combining food production with energy generation [104]. These systems use agricultural byproducts to produce bioenergy, while the residual heat and waste from energy production are used to enhance agricultural productivity [104]. This closed-loop system maximizes resource efficiency, which is an essential feature in displacement settings where resources are limited. Another effective strategy is establishing community seed banks, which preserves genetic diversity and provides access to locally adapted seeds [105,106]. These seed banks can serve as a crucial resource for climate refugees by ensuring seed availability in the wake of climatic disturbances or displacement [105,106]. They also play a role in maintaining agricultural biodiversity, which is key to resilience and adaptability. The integration of these alternative strategies can substantially improve food security for climate refugees, ensuring not just survival but sustainable development.

### 3.10. Policy Implications and Recommendations

The challenge of managing climate-induced migration involves not only addressing immediate needs but also strategizing for long-term resilience and sustainability. Drawing on recent studies and lessons from past policy experiences, we propose a set of recommendations aimed at more effectively supporting climate refugees. First, it is crucial to invest in training for local populations in vulnerable regions, particularly those frequently affected by climate variability such as erratic rainfall and crop failures. Providing skills for alternative livelihoods can help communities adapt to changing environmental conditions without the need to migrate [23]. Second, community gardens and kitchens are vital food security initiatives that empower communities, enhance self-reliance, and improve nutritional intake. Policies should support the establishment of community gardens with resources for dedicated groups and knowledgeable facilitators who understand local agricultural practices and climatic conditions [107]. Recent evidence also suggests that home gardens significantly enhance household food security and wellbeing by improving dietary diversity and promoting biodiversity, as demonstrated across diverse rural and low-income settings [108,109,110]. Similarly, community kitchens can be promoted to improve cooking skills and provide nutritious meals, serving as a sustainable alternative to food banks and aid dependency [111]. Third, experience has shown that policies designed to restrict migration are often counterproductive, exacerbating the challenges faced by refugees and the communities they move from and to [60,112]. Therefore, policies should facilitate the safe and legal movement of people, helping to distribute and mitigate the socio-economic impacts of migration rather than compounding them. Fourth, implementing principles of the circular economy can significantly contribute to the resilience of regions prone to natural disasters [113]. This includes initiatives to reduce waste, enhance resource efficiency, and innovate in sustainable food production and consumption. Such practices not only reduce environmental impact but also build economic resilience by creating jobs and sustainable growth in climate-vulnerable regions [114]. Fifth, continued investment is needed in the adaptive capacities of both origin and destination communities. This includes infrastructure improvements, sustainable agricultural practices, accessible healthcare, and comprehensive disaster preparedness programs [60,115,116]. These measures can mitigate the immediate impacts of climate events and reduce the long-term necessity for migration. Finally, supporting community-based initiatives that focus on capacity-building interventions and empower local populations to manage the impacts of climate change effectively through local projects that enhance food security, water conservation, and disaster preparedness [117,118].

Strengths and Limitations: The review conducted a thorough search across multiple renowned databases like Ovid MEDLINE, Embase, and others. This wide-ranging search ensured a comprehensive collection of relevant literature covering various aspects of food security and climate-induced migration. Moreover, adherence to the PRISMA-ScR guidelines strengthens the review’s rigour by ensuring that each step is conducted in a transparent and systematic manner. Further, by including studies with varied methodological approaches ranging from mixed methods to qualitative designs, the review encompassed a broad spectrum of perspectives and data, enriching the understanding of the complex phenomena under study. Despite these strengths, the review faces certain limitations. One major constraint is the language and accessibility of sources; by restricting the inclusion to articles available in English, potentially valuable insights from non-English sources, particularly from regions severely affected by climate issues, are overlooked. This limitation might skew the review’s comprehensiveness and its applicability across different global contexts. Additionally, the exclusion of grey literature and less formal sources like news articles and conference proceedings might omit the emerging trends not captured in peer-reviewed journals.

## 4. Conclusions

The complex relationship between climate change, migration, and food security presents significant challenges that demand strategic responses. The research reviewed highlights the varied impacts of climate variability on different communities, which highlights the need for adaptive strategies that are both resilient and context-specific. Migration, serving as both a coping mechanism and an adaptive response, varies greatly in effectiveness based on the socio-economic resilience and vulnerabilities of affected populations. Key insights from the studies emphasize the importance of targeted interventions to address the specific vulnerabilities of climate-impacted populations. These interventions should focus on enhancing local agricultural practices, improving urban food systems, and supporting migrants through better integration policies and robust support networks. Such measures can help mitigate the adverse effects of climate change and enhance food security for displaced populations and their host communities. Furthermore, the research highlights the critical role of community-based support and humanitarian aid in addressing immediate food security challenges. Innovative initiatives, such as community-based agroecological projects, CSA practices, and circular economy concepts, are essential in fostering sustainable food systems that can withstand climatic shocks. Policy recommendations call for a holistic approach that aligns migration policies with local development strategies. This approach should facilitate safe and legal migration while enhancing the adaptive capacities of both origin and destination communities. It should also integrate economic, social, and environmental sustainability to ensure comprehensive resilience against future climate impacts.

In conclusion, addressing the challenges posed by climate change, migration, and food security requires a coordinated effort from global to local levels, which involves a range of stakeholders from policymakers to local communities. By adopting adaptive, integrated, and forward-thinking strategies, we can better support climate refugees and vulnerable populations, ensuring their food security and overall well-being in the face of escalating climate risks. Future research should prioritize the development and use of standardized food security indicators, clearer and more consistent classifications of climate-related mobility, and rigorous evaluation of food security initiatives across different stages of displacement. Greater attention to destination food environments, longitudinal outcomes, and comparative assessments of intervention effectiveness is needed to strengthen the evidence base and support more targeted, sustainable responses to climate-related displacement.

## Data Availability

No new data were created or analyzed in this study. Data sharing is not applicable to this article.

## Abbreviations

The following abbreviations are used in this manuscript:

PRISMA-ScR	Preferred Reporting Items for Systematic Reviews and Meta-Analyses Extension for Scoping Reviews
IDPs	Internally Displaced Persons
PRA	Participatory Research Approach
CSA	Climate-smart Agriculture
IFES	Integrated Food Energy Systems

## Appendix A

**Table A1:** Example of the search strategy.

1	Refugee camp/OR refugee/OR refugee*
2	“Climate change” OR “global warming” OR “greenhouse effect” OR “climate adaptation” OR “extreme weather” OR “climate crisis”
3	“Food secur*” OR “Food insecur*” OR “Food Safety” OR “Food Sufficiency” OR “Nutrition security” OR “Food supply” OR “Food availability” OR “Food access” OR “Food utilization” OR “Food management” OR “Food affluence”
4	1 and 2 and 3
